# Direct Acting Oral Anticoagulant vs. Warfarin in the Prevention of Thromboembolism in Patients With Non-valvular Atrial Fibrillation With Valvular Heart Disease—A Systematic Review and Meta-Analysis

**DOI:** 10.3389/fcvm.2021.764356

**Published:** 2021-12-16

**Authors:** Januar Wibawa Martha, Raymond Pranata, Wilson Matthew Raffaelo, Arief Wibowo, Mohammad Rizki Akbar

**Affiliations:** ^1^Department of Cardiology and Vascular Medicine, Faculty of Medicine Universitas Padjadjaran, Rumah Sakit Umum Pusat Hasan Sadikin, Bandung, Indonesia; ^2^Faculty of Medicine, Universitas Pelita Harapan, Tangerang, Indonesia

**Keywords:** DOAC, direct-acting oral anticoagulant, NOAC, vitamin K antagonist (VKA), valvular heart disease, atrial fibrillation

## Abstract

**Purpose:** There is uncertainty as to which anticoagulant should be used in non-valvular atrial fibrillation (AF) with valvular heart disease. This systematic review and meta-analysis aimed to assess the efficacy and safety of direct-acting oral anticoagulants (DOACs) compared with warfarin in patients with non-valvular AF with valvular heart disease.

**Methods:** We performed a comprehensive literature search using PubMed, Scopus, Embase, and Clinicaltrials.gov from the inception of databases up until August 2, 2021, and the search was updated and finalized on October 17, 2021. The intervention group was DOACs and the control group was warfarin. The primary outcome was systemic embolism and stroke (SSE), and the secondary outcome was major bleeding and intracranial hemorrhage. The pooled effect estimate was reported as the hazard ratio (HR) and odds ratio (OR).

**Results:** There were 21,185 patients from seven studies included in this systematic review and meta-analysis. Stroke and systemic embolism were lower in patients receiving DOACs [HR 0.76 (95% CI 0.67, 0.87), *p* < 0.001; I^2^: 5%] compared with warfarin. The subgroup analysis on RCTs showed the significant reduction of SSE in the DOACs group [HR 0.73 (95% CI 0.60, 0.89), *p* = 0.002; I^2^: 16%]. There was no significant difference in terms of major bleeding [HR 0.89 (95% CI 0.75, 1.05), *p* = 0.18; I^2^: 69%]. Intracranial hemorrhage [HR 0.42 (95% CI 0.22, 0.80), *p* = 0.008; I^2^: 73%] were lower in the DOAC group.

**Conclusion:** This meta-analysis indicates that DOACs were associated with a lower risk of SSE and intracranial hemorrhage compared with patients receiving warfarin. There was no significant difference between the two groups in terms of major bleeding.

## Introduction

One of the most important complications of atrial fibrillation (AF) is a systemic embolism, mainly stroke. The risk can be mitigated by lifelong anticoagulation; however, anticoagulation predisposes patients to bleeding. Thus, long-term efficacy and safety are important to balance the risk and benefit of anticoagulation (1). Due to its more predictable pharmacodynamic, safety profile, and lack of required monitoring, direct oral anticoagulants (DOACs) are more favorable than vitamin K antagonist which needs more routine monitoring (2). However, there is uncertainty as to which anticoagulant should be used in non-valvular AF with valvular heart disease (3).

Recent studies compared the use of DOACs vs. warfarin in patients with non-valvular AF with valvular heart disease, aiming to resolve this issue. This systematic review and meta-analysis aimed to assess the efficacy and safety of DOACs compared to warfarin in patients with non-valvular AF with valvular heart disease.

## Methods

This systematic review follows the reporting guideline of the Preferred Reporting Items for Systematic Reviews and Meta-analysis (PRISMA).

### Search Strategy and Study Selection

We performed a comprehensive literature search using PubMed, Scopus, Embase, and Clinicaltrials.gov using the keywords “(rivaroxaban OR Xarelto OR dabigatran OR apixaban OR edoxaban) AND (warfarin OR vitamin K antagonist or coumadin) AND (atrial fibrillation) AND (valvular heart disease)” from the inception of databases up until August 2, 2021, and the search was updated and finalized on October 17, 2021. Two independent authors screened the title and abstracts for eligibility based on the inclusion and exclusion criteria. Discrepancies that arose were resolved by discussion.

### Inclusion and Exclusion Criteria

The population was patients with non-valvular AF with valvular heart disease which is defined as the presence of AF in the absence of moderate-to-severe mitral stenosis or a mechanical heart valve, with concomitant aortic stenosis/regurgitation, tricuspid valve stenosis/regurgitation, pulmonic stenosis/regurgitation, mitral regurgitation, mitral valve prolapse, bioprosthetic valve, or valve repair. The intervention group was patients receiving DOACs and the control group was patients receiving warfarin. The primary outcome was systemic embolism and stroke (SSE), defined as systemic embolism and stroke as a result of cardiac embolism. The secondary outcome was major bleeding and intracranial hemorrhage.

Studies that met the following criteria were included: (1) observational studies or randomized controlled trials evaluating patients with non-valvular AF and valvular heart disease, (2) comparing DOACs and warfarin, and (3) primary and/or secondary outcomes.

Studies that met at least one of the following criteria were excluded: (1) non-research letters, (2) abstract-only publication, (3) reviews, and (4) commentaries or editorial. There was no language restriction imposed.

### Data Extraction

Data extraction from the included studies was performed by two independent authors. The first author, study design, sample size, valvular involvement, age, sex, hypertension, diabetes, coronary artery disease, paroxysmal AF, CHADS2-VASc, HAS-BLED, and the primary and secondary outcomes were the data of interest. Discrepancies that arose were resolved by discussion.

### Risk of Bias Assessment

The Newcastle Ottawa Scale (NOS) for cohort studies was used to assess the observational studies and the Cochrane risk of bias assessment tool was used to assess the randomized controlled trials (4). The NOS comprised three domains: (1) selection, (2) comparability, and (3) outcome of the included studies. The assessment was performed by two independent authors and discrepancies were resolved by discussion.

### Outcome

The primary outcome was SSE, defined as systemic embolism and stroke as a result of the cardiac embolism. The secondary outcome was major bleeding and intracranial hemorrhage. The pooled effect estimate was reported as hazard ratio (HR) and odds ratio (OR).

### Statistical Analysis

The HR and dichotomous data containing events per total of intervention and control group were extracted from each study. The log HR and standard error were then calculated and pooled using the random-effects inverse-variance method. The Mantel-Haenszel method was used to calculate the ORs using the random-effects model regardless of heterogeneity for dichotomous values. *P*-values were considered statistically significant if they were below 0.05. The Cochran's *Q*-test and I^2^ statistics were used to assess heterogeneity; I^2^ values above 50% or/and *p*-value below 0.10 indicated statistically significant heterogeneity. The Funnel-plot analysis and Egger's test were used to assess publication bias and small-study effects. Review Manager 5.4 and STATA 16.0 were used to perform the meta-analysis.

## Results

### Baseline Characteristics

**Figure 1:**
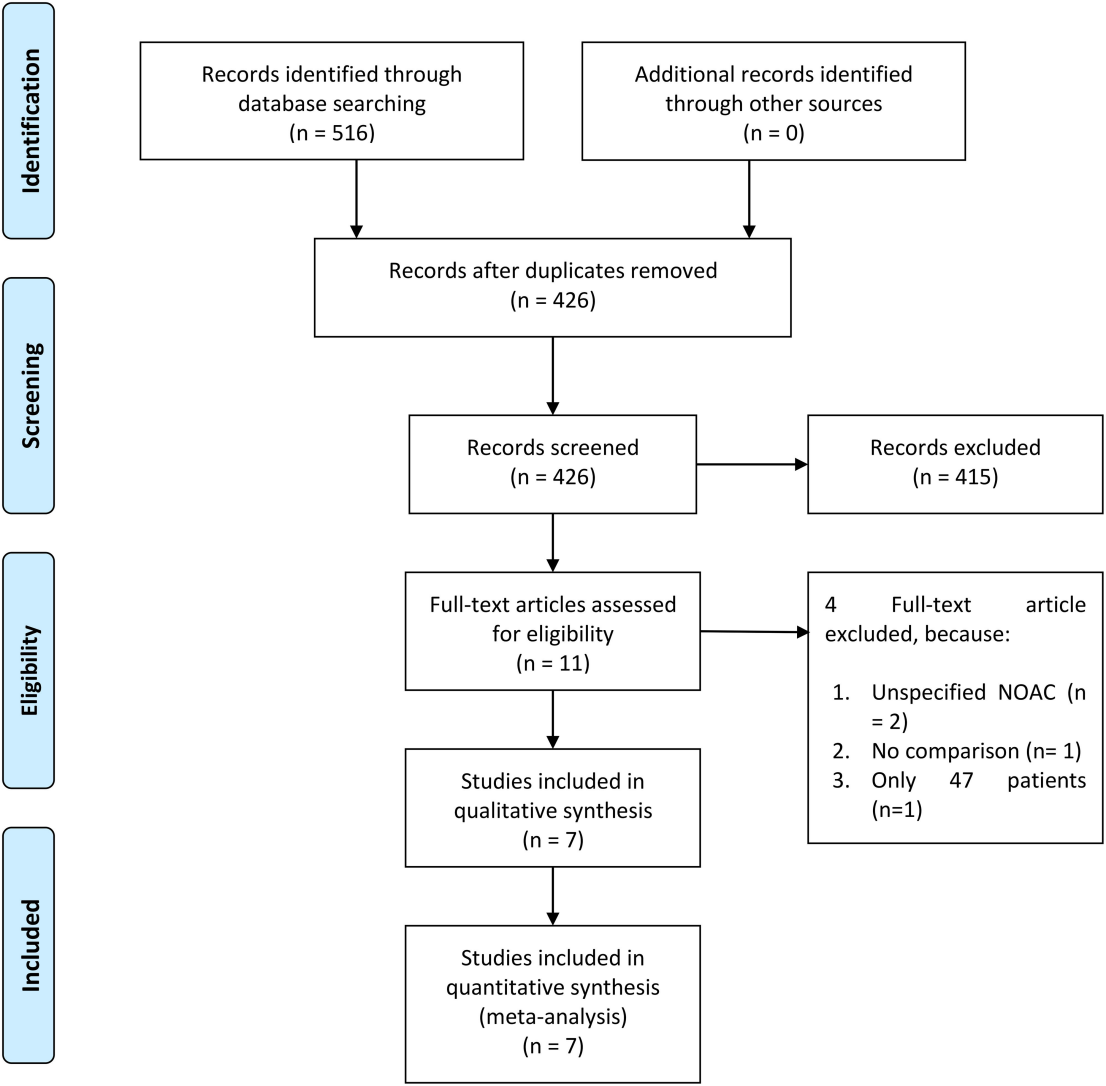
Preferred reporting items for systematic reviews and meta-analysis (PRISMA) flowchart.

There were 21,185 patients from seven studies included in this systematic review and meta-analysis (Figure 1) (5–11). Different types of DOACs were included in the study.

There were four studies by Breithardt et al., Briasoulis et al., Guimarães et al., and Strange et al. that compared rivaroxaban to warfarin. Two studies compared dabigatran to warfarin, namely Briasoulis et al. and Ezekowitz et al. There was one study by Avezum et al. that compared apixaban to warfarin. There were two observational studies (one propensity-score matched) and five randomized controlled trials. Out of five randomized controlled trials, one was open-label and four were double-blinded. The baseline characteristics of the included studies can be seen in Table 1.

**Table 1:** Baseline characteristics of the included studies.

**References**	**Design**	**DOAC**	**Location**	**Sample**	**Valve**	**Age (years)**	**Male** **(%)**	**Smoker** **(%)**	**Hypertension (%)**	**Diabetes** **(%)**	**HF** **(%)**	**Paroxysmal** **AF** **(%)**	**CHA2DS2-VASc**	**HAS-BLED**	**Primary** **(%)**	**Secondary** **(%)**	**GI** **bleeding** **(%)**	**NOS**
Breithardt et al. (8)	RCT (ROCKET AF)	Rivaroxaban	United States	1,992	AS 11% AR 24.8% MR 89.6% Other 0.6% Prosthetic excluded	75	61	39	91	40	70	16	3.5	2.8	9.2	17.0	3.5	Figure 6
Briasoulis et al. (6)^*^	PSM 1:1 Observational	Rivaroxaban and Dabigatran	United States	18,137 (5,871)	NA, Bioprosthetic excluded	77	46	NA	86	37	27	NA	4.5	1.8	1.8	4.8	2.1	8
Guimarães et al. (5)	RCT (RIVER Trial)	Rivaroxaban	Brazil	1,005	Bioprosthetic MV	59	40	4	61	14	39	22	2.6	1.6	3.2	4.1	NA	Figure 6
Strange et al. (7)	Observational	Rivaroxaban	Denmark	1,735	AS 61% AR 22.2% Bioprosthetic 19.4% MR 30.3%	79	54	NA	74	13	29	29	3.5	2.6	5.2	11.2	NA	8
Avezum et al. (9)	RCT (ARISTOTLE)	Apixaban		4,808	AS 8% AR 18.4% MR 73.3% Number of bioprosthetic valve was unknown	71	59	NA	85.3	22.6	48.6	12.4	2.2	NA	3.2	4.6	NA	8
Ezekowitz et al. (11)	RCT (RE-LY)	Dabigatran		3,950	AS 12% AR 21% MR 79% Prosthetic excluded	74	59	NA	77	24	60.3	NA	2.0	NA	1.61/years	4.36/years	NA	8
De Caterina et al. (10)	RCT (ENGAGE AF–TIMI 48)	Edoxaban		2,824	AS 6% AR 13% MR 80% Bioprosthetic 6.8%	71.8	58	NA	93	32	73.7	19.7	4.56	2.55	1.79/years	3.16/years	2.1	8

* *Characteristics was for before PSM*.

*AF, Atrial Fibrillation; AS, Aortic Stenosis; AR, Aortic Regurgitation; DOAC, Direct Acting Anticoagulant; GI, Gastrointestinal; HF, Heart Failure, MR, Mitral Regurgitation; MV, Mitral Valve; NOS, Newcastle-Ottawa Scale; PSM, Propensity-Score Matching; RCT, Randomized Controlled Trial*.

### Systemic Embolism

**Figure 2:**
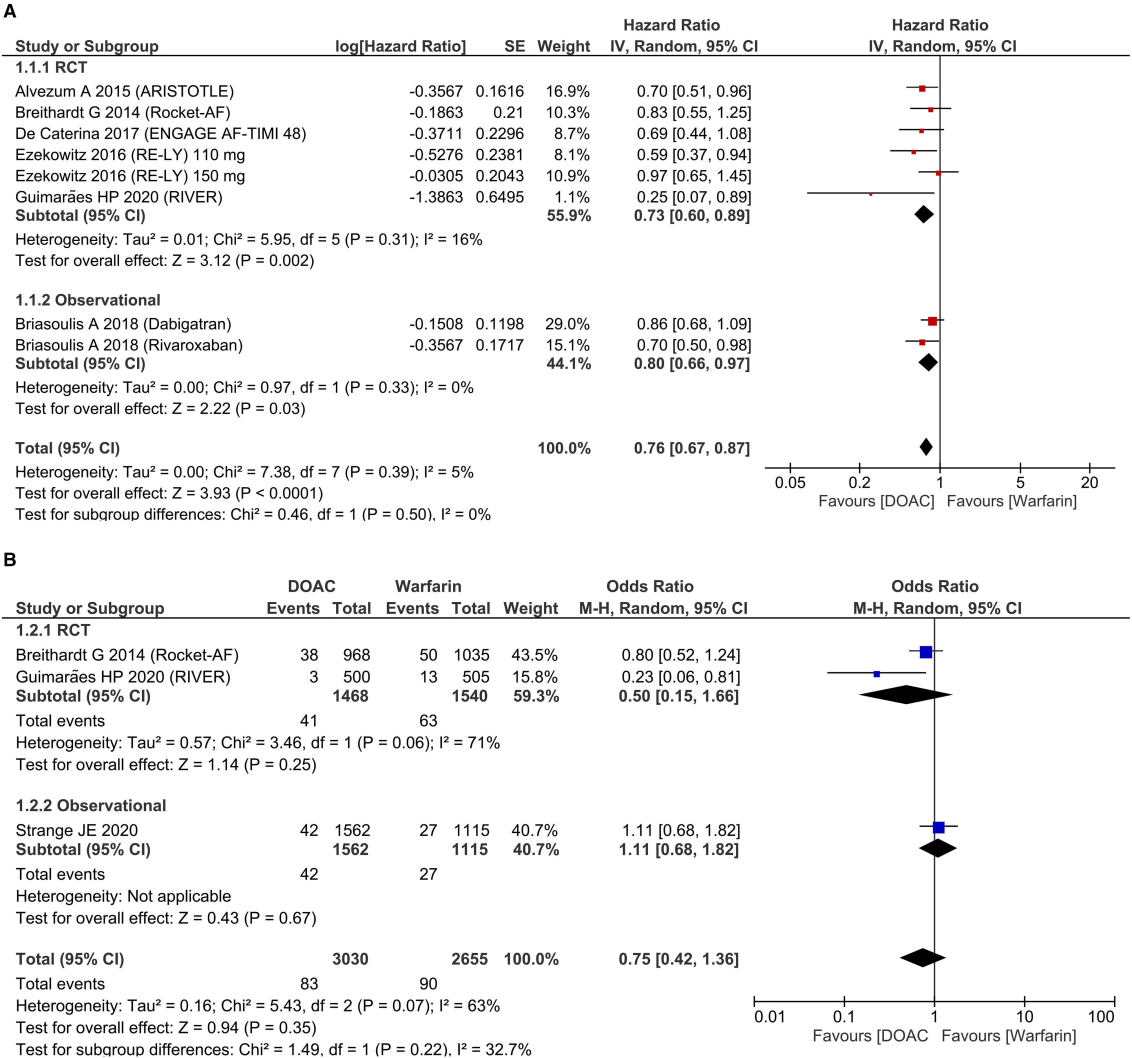
Systemic embolism and stroke. Pooled hazard ratio **(A)** and dichotomous outcome **(B)**.

Stroke and systemic embolism were lower in patients receiving DOAC [HR 0.76 (95% CI 0.67, 0.87), *p* < 0.001; I^2^: 5%, *p* = 0.39] compared to warfarin (Figure 2A). The subgroup analysis on RCTs showed the significant reduction of SSE in the DOAC group [HR 0.73 (95% CI 0.60, 0.89), *p* = 0.002; I^2^: 16%, *p* = 0.31]. For dichotomous outcomes, pooled analysis did not show significant difference in terms of SSE [OR 0.75 (95% CI 0.42, 1.36), *p* = 0.35; I^2^: 63%, *p* = 0.07] (Figure 2B).

### Bleeding

**Figure 3:**
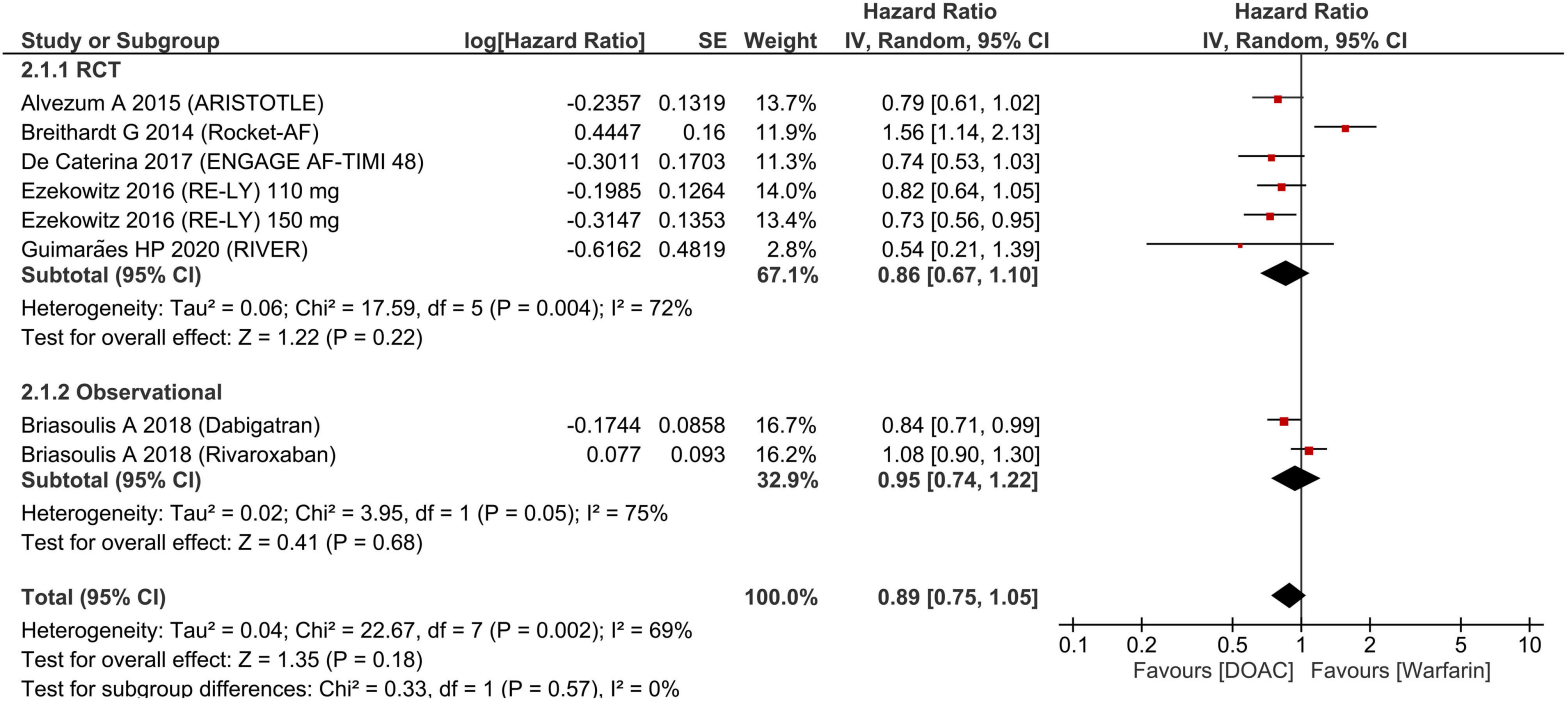
Major bleeding.

**Figure 4:**
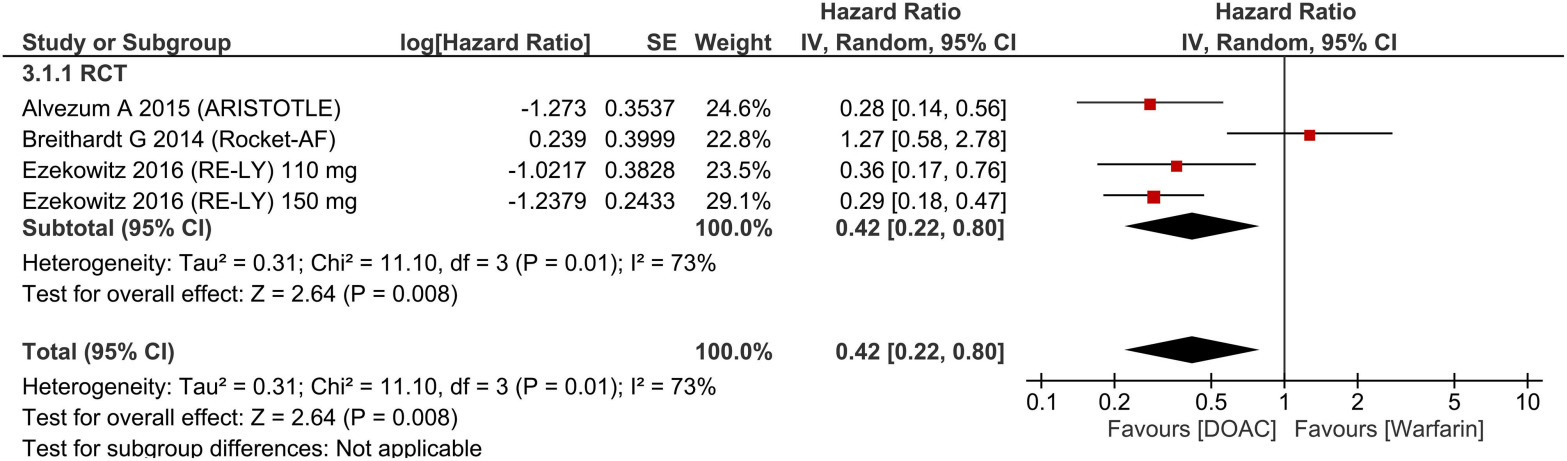
Intracranial hemorrhage.

There was no significant difference in terms of major bleeding [HR 0.89 (95% CI 0.75, 1.05), *p* = 0.18; I^2^: 69%, *p* = 0.002] (Figure 3). Intracranial hemorrhage (HR 0.42 (95% CI 0.22, 0.80), *p* = 0.008; I^2^: 73%, *p* = 0.001] (Figure 4) were lower in the DOAC group. For dichotomous outcomes, pooled analysis did not show significant difference in terms of major bleeding [OR 0.94 (95% CI 0.56, 1.57), *p* = 0.82; I^2^: 74%, *p* = 0.02] and intracranial hemorrhage [OR 1.29 (95% CI 0.38, 4.35), *p* = 0.68; I^2^: 75%, *p* = 0.02].

### Publication Bias

**Figure 5:**
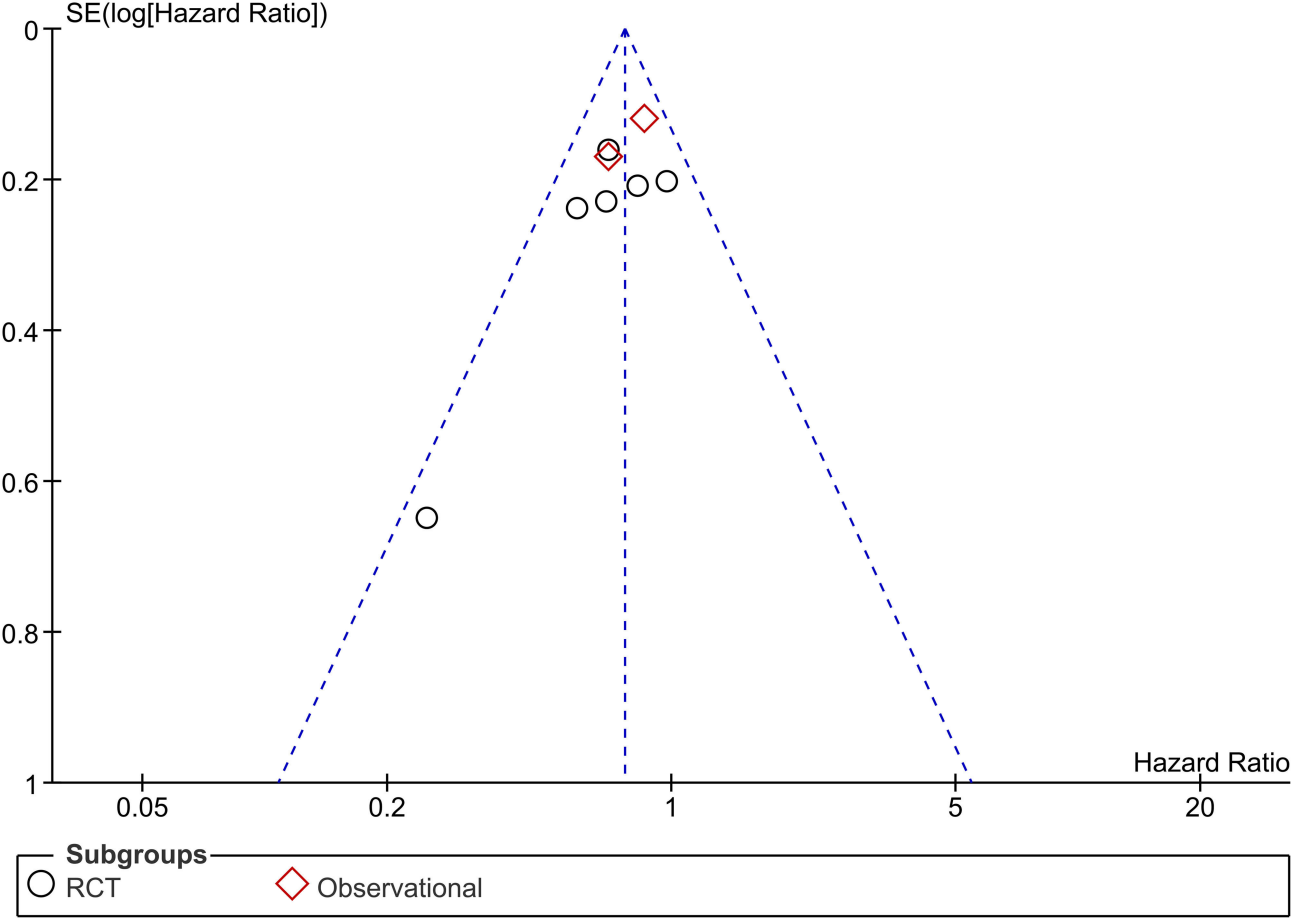
Funnel-plot analysis.

**Figure 6:**
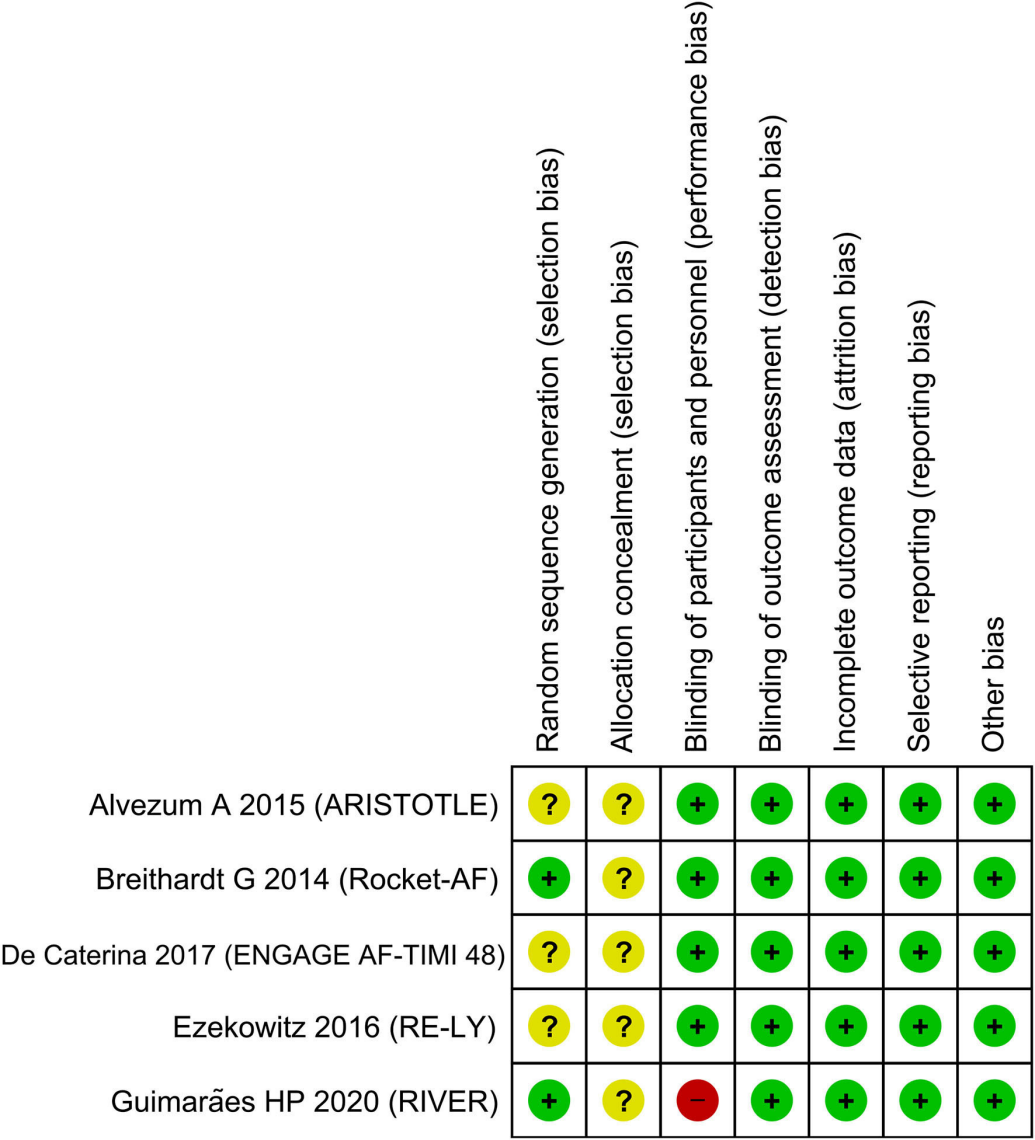
Risk of bias assessment.

The funnel plot was asymmetrical (Figure 5) and the Egger's test indicated that there was no indication of small-study effects (*p* = 0.420) for the pooled effect estimate of the primary outcome. The risk of bias is based on the Cochrane RoB tool can be seen in Figure 6.

## Discussion

This meta-analysis indicates that DOACs were associated with a lower risk of SSE and intracranial hemorrhage compared to patients receiving warfarin. There was no significant difference between the two groups in terms of major bleeding. Thus, either DOAC or warfarin may be used depending on the patient's profile.

Warfarin has been long known to reduce the risk of ischemic stroke in patients with atrial fibrillation. However, frequent monitoring and dose adjustment are needed and might be inconvenient and not suitable for the patient (12). Recently, the use of DOACs has been approved for stroke prevention in patients with non-valvular atrial fibrillation (2, 13). DOACs are preferred in daily practice over Vitamin K Antagonist (VKA) mainly due to their better safety profile and lack of required monitoring (12). However, the scarcity of evidence remains an issue due to the small proportion of trials, conflicting, or unexplainable results. For instance, the efficacy and safety of oral anticoagulants seem to differ in patients with aortic stenosis (AS) in comparison to other valvular heart diseases such as mitral regurgitation or aortic regurgitation (14). Breithardt et al. concluded that the use of rivaroxaban in patients with non-valvular AF patients with mitral regurgitation or aortic regurgitation might increase the risk of major bleeding in comparison to those who received warfarin (14). In contrast, Guimarães et al. reported that the incidence of stroke in patients who received rivaroxaban was slightly lower in comparison to those who received warfarin in patients with bioprosthetic mitral-valve surgery (5). In addition, both agents also showed a similar rate of bleeding and valve thrombosis (5).

The underlying valvular pathology might also contribute to the disturbance of hemostasis. The change in platelet indices and turbulent flow contribute to the activation of the coagulation cascade (15–17). The mild calcification of the aortic valve exhibits anti-aggregatory effects while severe aortic stenosis does not demonstrate the same effects (18). AS also demonstrated a decreased level of von Willebrand factor while in contrast, AF itself is related to higher levels of von Willebrand factor than AF absence (19, 20). Therefore, these two mechanisms contribute to the opposing effects in coagulation system disturbance.

While DOACs offer convenience, more predictable dynamics, and relatively less interaction with other drugs that the patient might be taking in comparison to VKA, the choice of using DOAC or VKA has to be tailored to each of the patients since the outcome of DOACs and VKA might differ depending on the patient's clinical profile and underlying valvular heart disease. The patient's kidney and liver baseline status might also be taken into consideration before prescribing DOAC or VKA. DOACs might offer slightly better outcomes related to thromboembolism and bleeding in comparison to warfarin (5, 21). Meanwhile, rivaroxaban might have to be used with caution because of its slightly increased risk of bleeding in patients with AS, AR, and MR (14). In conclusion, while both drugs might be used as a prevention against thrombotic or embolic events, they are associated with different outcomes across the underlying valvular pathology and the choice between DOACs or VKA has to be adjusted according to the patient's clinical profile.

The limitation of this meta-analysis is that only two studies were randomized controlled trials. The number of studies is too small to perform adequately powered meta-regression analysis, thus we cannot analyze whether a certain type of valvular heart disease or prosthetic valve will correspond to a better outcome with a certain anticoagulant. Due to inadequate data to perform subsequent analysis for specific types of valve disease, we cannot explore whether the types of valvular heart disease are the cause of heterogeneity in this study. In order to extensively analyze this aspect, more studies are needed and the meta-analysis of individual participant data is required. The weight among the studies included in this meta-analysis was not equal.

## Conclusion

This meta-analysis indicates that DOACs were associated with a lower risk of SSE and intracranial hemorrhage compared to patients receiving warfarin in patients with non-valvular AF with valvular heart disease. There was no significant difference between the two groups in terms of major bleeding. Thus, either DOAC or warfarin may be used depending on the patient's profile.

## Data Availability Statement

The original contributions presented in the study are included in the article/supplementary material, further inquiries can be directed to the corresponding author/s.

## Author Contributions

JM: conceptualization, investigation, writing—review and editing, and supervision. RP: conceptualization, methodology, software, data curation, formal analysis, investigation, validation, writing—original draft, and writing—review and editing. WR: data curation, investigation, and writing—original draft. AW: investigation and writing—original draft. MA: investigation and writing—review and editing. All authors contributed to the article and approved the submitted version.

## Conflict of Interest

The authors declare that the research was conducted in the absence of any commercial or financial relationships that could be construed as a potential conflict of interest.

## Publisher's Note

All claims expressed in this article are solely those of the authors and do not necessarily represent those of their affiliated organizations, or those of the publisher, the editors and the reviewers. Any product that may be evaluated in this article, or claim that may be made by its manufacturer, is not guaranteed or endorsed by the publisher.
